# Paving the Way: a Systematic Review and Meta-Analysis of Randomized Controlled Trials of Atherectomy as Vessel Preparation Prior to Balloon Angioplasty in Peripheral Arterial Disease

**DOI:** 10.1007/s00270-026-04527-y

**Published:** 2026-07-13

**Authors:** Joshua Qi Jing Ho, Sonal Udayasiri, Michael Stewart, Andy Owen, Numan Kutaiba, Jessie Low, Ali Khabaza, Dinesh Ranatunga, Julian Maingard, Mark Brooks, Ashu Jhamb, Lucy Corlette, Charles Handley, Christen Barras, Hong Kuan Kok, Hamed Asadi

**Affiliations:** 1 Melbourne Health, Parkville, VIC Australia; 2 Department of Radiology, Austin Health, Heidelberg, VIC Australia; 3 Department of Interventional Radiology, Austin Health, Heidelberg, VIC Australia; 4 Department of Neurointerventional Radiology, St. Vincent’s Hospital, Fitzroy, VIC Australia; 5 Department of Neurointerventional Radiology, Austin Health, Heidelberg, VIC Australia; 6 Department of Radiology, Royal Melbourne Hospital, Melbourne, Australia; 7 Department of Radiology, Monash Health, Clayton, VIC Australia; 8 Department of Radiology, Royal Adelaide Hospital, Adelaide, Australia; 9 South Australian Health and Medical Research Institute, Adelaide, Australia; 10 Adelaide University, Adelaide, Australia; 11 Interventional Radiology Service, Northern Imaging Victoria, Northern Health, Melbourne, Australia; 12 NECTAR Research Group, Northern Health, Melbourne, Australia; 13 Faculty of Medicine, Dentistry and Health Sciences, University of Melbourne, Melbourne, Australia; 14 School of Health and Biomedical Sciences, RMIT University, Melbourne, Australia; 15 Department of Neurointerventional Radiology, Monash Health, Clayton, VIC Australia; 16 School of Medicine, Deakin University, Warun Ponds, Geelong Australia

**Keywords:** Peripheral arterial disease, Atherectomy, Balloon angioplasty, Vessel preparation, Drug-coated balloon

## Abstract

**Purpose:**

This systematic review and meta-analysis compares the safety and efficacy of atherectomy as a vessel preparation tool prior to balloon angioplasty (ATH + BA) versus balloon angioplasty alone (BA) for the treatment of peripheral arterial disease (PAD).

**Materials and Methods:**

Medline, Embase, CENTRAL, and Clinicaltrials.gov databases were searched for randomized controlled trials (RCTs) comparing ATH + BA versus BA alone in treating PAD. The outcomes analysed included primary patency, technical success, bailout stenting, flow-limiting dissection, clinically driven target lesion revascularization (CD-TLR), distal embolization, arterial perforation, major amputation, and all-cause mortality. A subgroup analysis was done to compare femoropopliteal versus infrapopliteal and plain balloon angioplasty versus drug-coated balloon.

**Results:**

Seven RCTs met the inclusion criteria (489 patients). Atherectomy as vessel preparation was associated with reduced rates of bailout stenting (OR: 0.14, 95% CI 0.03–0.67, *p* = 0.01) and flow-limiting dissection (OR: 0.29, 95% CI 0.10–0.84, *p* = 0.02). Primary patency (OR: 2.13, 95% CI 1.00–4.50, *p* = 0.049) and CD-TLR at 6 months (OR: 0.24, 95% CI 0.06–0.96, *p* = 0.04) results favoured ATH + BA. No significant differences were found in technical success, 12-month primary patency, CD-TLR at 12 months, distal embolization, arterial perforation, major amputation, or all-cause mortality. Subgroup analysis demonstrated a reduction in bailout stenting in femoropopliteal studies (OR: 0.05, 95% CI 0.01–0.21) compared with infrapopliteal studies (OR: 0.86, 95% CI 0.31–2.40).

**Conclusions:**

This meta-analysis found that atherectomy as vessel preparation prior to BA reduced bailout stenting, flow-limiting dissections, and 6-month CD-TLR, with improved 6-month patency compared with BA alone. However, these benefits did not translate into improvements in clinical outcomes at 12 months. Larger RCTs are needed to establish the clinical benefit of atherectomy.

**Graphical Abstract:**

**Figure:**
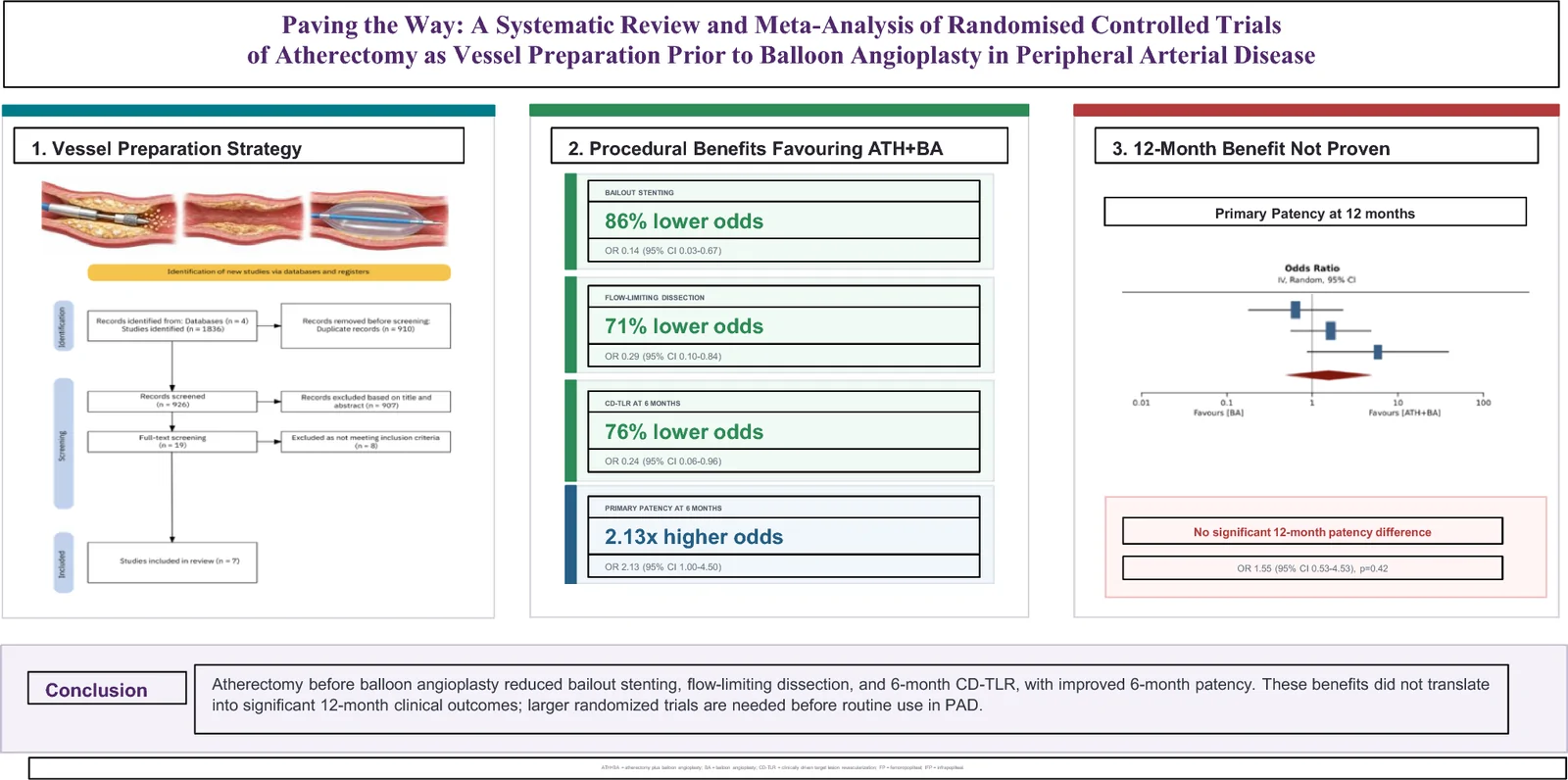

**Supplementary Information:**

The online version contains supplementary material available at https://doi.org/10.1007/s00270-026-04527-y.

## Introduction

Peripheral arterial disease (PAD) is a major manifestation of systemic atherosclerosis, affecting over 230 million people worldwide [1]. Clinical manifestations range from intermittent claudication to chronic limb-threatening ischaemia (CLTI) and carry substantial risks of amputation and death [2].

Endovascular revascularization has emerged as first-line treatment for symptomatic PAD. Plain balloon angioplasty (PBA) works by mechanically displacing or remodelling plaque to improve luminal calibre, but its limitations include issues with elastic recoil, flow-limiting dissection, and restenosis [3, 4]. Drug-coated balloons (DCBs) have improved femoropopliteal (FP) outcomes and restenosis rates by delivering antiproliferative agents to reduce neointimal hyperplasia [5]. However, treatment of heavily calcified atherosclerotic lesions remains challenging with reduced primary technical success rates due to dissection, recoil and subsequent increased bailout stenting rates [3, 5].

Atherectomy technology consists of catheter-based devices that actively remove and reduce atherosclerotic plaque burden [6]. Early studies evaluated atherectomy as a standalone alternative, testing whether plaque excision alone could replace balloon dilatation. In contrast, contemporary practice positions atherectomy as an adjunct for vessel preparation performed prior to balloon angioplasty, facilitating a “leave-nothing-behind” approach [7]. These represent distinct clinical questions; the former asks whether atherectomy is better than balloon angioplasty, while the latter asks whether adding atherectomy improves outcomes beyond BA alone.

This systematic review and meta-analysis evaluates the efficacy and safety of atherectomy as vessel preparation prior to BA compared with BA alone in adults with symptomatic PAD.

## Materials and Methods

### Literature Search

This systematic review and meta-analysis was conducted in accordance with the Preferred Reporting Items for Systematic Reviews and Meta-Analyses (PRISMA) 2020 statement [8]. A comprehensive literature search was performed across four electronic databases: MEDLINE (via Ovid, 1946 to September 22, 2025), Embase (via Ovid, 1974 to September 22, 2025), Cochrane Central Register of Controlled Trials (CENTRAL), and ClinicalTrials.gov.

The search strategy combined Medical Subject Headings (MeSH) terms and free-text terms covering PAD, anatomical locations, and atherectomy devices, with a filter for RCT applied. Full search strategies are provided in the Supplementary Material.

### Inclusion and Exclusion Criteria

#### Inclusion criteria

(1) RCTs comparing ATH + BA versus BA alone for the treatment of de novo atherosclerotic lesions in the FP or IFP arteries; (2) adult patients (≥ 18 years) with symptomatic PAD (Rutherford category 2–6); (3) adjunctive balloon type matched between intervention and comparator arms; (4) the study reported at least one of the prespecified endpoints; (5) published in English.

#### Exclusion criteria

(1) Non-randomized studies, including observational studies, cohort studies, case series, and case reports; (2) studies comparing atherectomy alone versus balloon angioplasty; (3) studies with unmatched balloon types between arms; (4) studies treating in-stent restenosis, acute limb ischaemia, or non-atherosclerotic disease.

### Quality Assessment

The risk of bias for included RCTs was assessed using the Cochrane Risk of Bias 2.0 (RoB 2) tool [9]. Each study was evaluated across five domains with each domain judged as either "low risk", "some concerns", or "high risk" of bias. An overall risk of bias judgement was assigned based on the judgments across all domains.

### Data Extraction

The studies were screened by two independent reviewers with title and abstract screen; any discrepancy in outcomes was assigned to a third reviewer for resolution. A full-text screen was subsequently conducted for eligible studies.

Data were extracted using a standardized data extraction form. For studies with multiple publications, data were consolidated and treated as an individual RCT. The primary outcome was primary patency at 12 months. Secondary outcomes included primary patency at 6 months, technical success, bailout stenting, flow-limiting dissection, distal embolization, arterial perforation, clinically driven target lesion revascularization (CD-TLR) at 6 and 12 months, major amputation, and all-cause mortality at 12 months. Outcome definitions for the above outcomes are provided in Supplementary Table 1.

### Statistical Analysis

Meta-analysis was performed using Python (NumPy, SciPy, and Matplotlib). Dichotomous outcomes were expressed as odds ratios (OR) with 95% confidence intervals (CI), calculated on a log scale and pooled by inverse-variance weighting, using a DerSimonian–Laird random-effects model, as there were anticipated clinical heterogeneity across devices, lesion locations, and balloon types. Forest plots were displayed in inverse-variance random-effects style consistent with RevMan format. The overall effect was assessed using the Z-test, with *p* < 0.05 considered statistically significant.

For studies with zero event cells, including double-zero studies, a continuity correction of 0.5 was added to all four cells of the 2 × 2 table, whilst studies without zero cells were analysed using uncorrected counts. Double-zero studies were retained to preserve information on outcomes with low event rates.

Heterogeneity among studies was assessed using Cochran's Q test (with *p* < 0.10 indicating significant heterogeneity). Between-study variance *τ*^2^ and the *I*^2^ statistic were interpreted as low (< 40%), moderate (40–60%), or substantial (> 60%). In subgroup analyses, *τ*^2^ was estimated separately within each subgroup.

## Results

### Study Selection and Characteristics

**Fig. 1:**
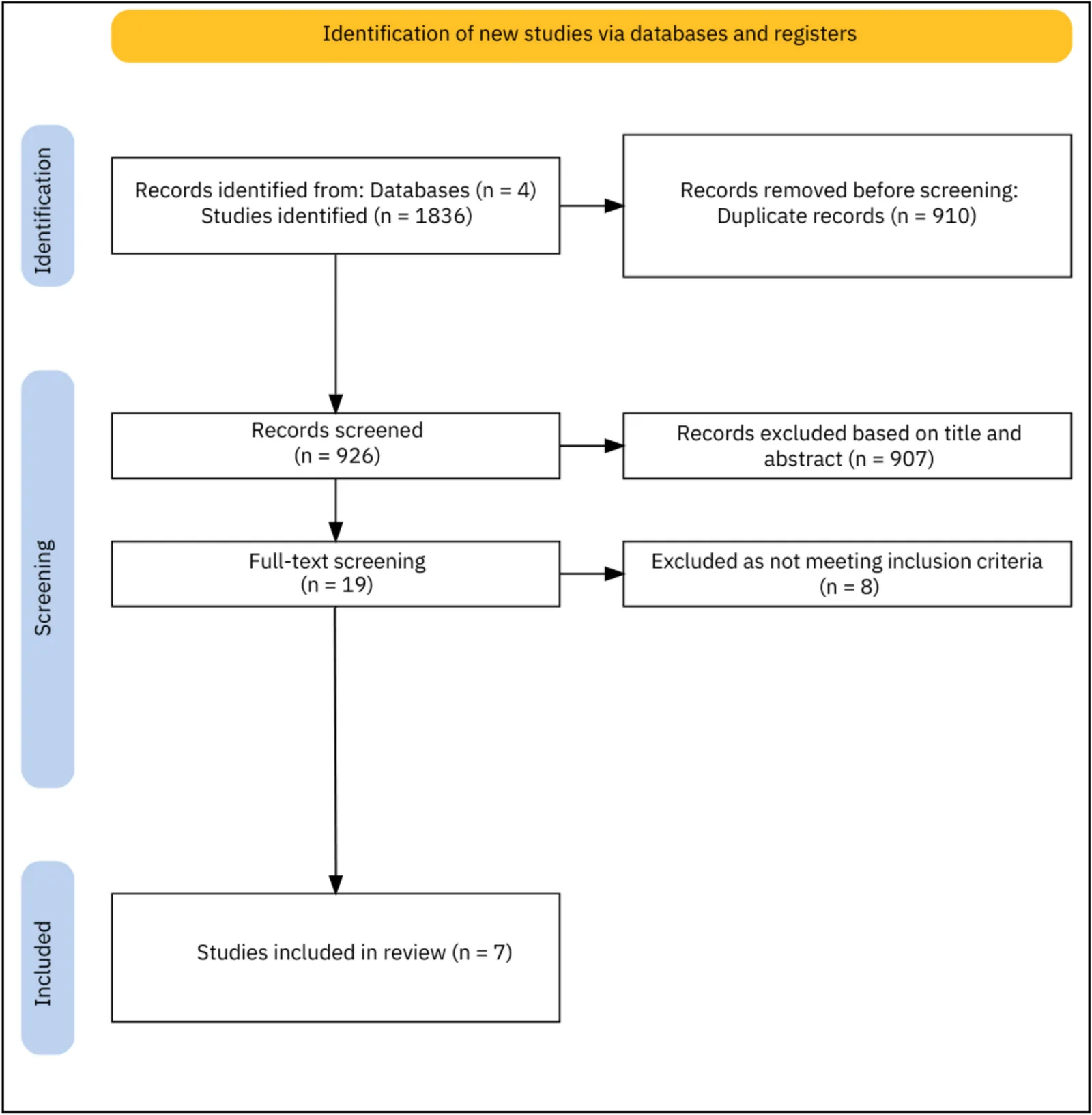
PRISMA diagram

The database search yielded 1,836 records across four databases (MEDLINE: 875, Embase: 660, CENTRAL: 37, ClinicalTrials.gov: 264). There were 910 duplicates removed, which yielded 926 unique records that underwent abstract screening. Of these, 907 were excluded as irrelevant, leaving 19 articles for full-text screening. Seven RCTs in the analysis [10–16] met all inclusion criteria and were included in the meta-analysis. The PRISMA flow diagram is presented in Fig. 1.

The baseline characteristics of the seven included RCTs are summarized in Table 1. All trials were published between 2012 and 2022, where four trials evaluated FP lesions [10–13], while three focused on infrapopliteal (IFP) disease [14–16]. Two trials used plain balloon angioplasty in both arms [10, 14], while five trials utilized drug-coated balloons [11–13, 15, 16]. Atherectomy modalities also varied and included directional and orbital atherectomy in three studies, respectively, and rotational atherectomy in one.

**Table 1:** Included studies in the systematic review

Author, year	Full article title
Shammas [14]	Six-month outcomes of prospective, randomized CALCIUM 360 study demonstrate the advantages of plaque modification with the orbital technology versus treatment with balloon angioplasty in patients with critical limb ischemia. Journal of the American College of Cardiology 2011;58(20 Suppl 1):B154
	Comparison of orbital atherectomy plus balloon angioplasty versus balloon angioplasty alone in patients with critical limb ischaemia: results of the CALCIUM 360 randomized pilot trial. Journal of Endovascular Therapy 2012;19(4):480–488
	Orbital atherectomy and balloon angioplasty versus balloon angioplasty alone in critical limb ischemia: results of the CALCIUM 360 trial. Journal of Vascular and Interventional Radiology 2013;24(1):145.e2-3
Dattilo [10]	The COMPLIANCE 360° Trial: a randomized, prospective, multicenter, pilot study comparing acute and long-term results of orbital atherectomy to balloon angioplasty for calcified femoropopliteal disease. Journal of Invasive Cardiology 2014;26(8):355–360
Zeller [11]	Directional atherectomy followed by a paclitaxel-coated balloon to inhibit restenosis and maintain vessel patency: twelve-month results of the DEFINITIVE AR study. Circulation: Cardiovascular Interventions 2017;10(9):e004848
	Directional atherectomy followed by paclitaxel-coated balloon angioplasty for the treatment of femoropopliteal artery disease: rationale and design of the DEFINITIVE AR study. Journal of Cardiovascular Surgery 2014;55(4):551–558
Cai [12]	Directional atherectomy combined with drug-coated balloon versus drug-coated balloon alone for treatment of femoropopliteal artery disease. Annals of Vascular Surgery2020;64:181–187
Rastan [15]	Atherectomy and drug-coated balloon angioplasty in treatment of long infrapopliteal lesions: rationale and design of a randomized controlled trial. Journal of Cardiovascular Surgery 2013;54(4):533–540
	Atherectomy and drug-coated balloon angioplasty for the treatment of long infrapopliteal lesions: a randomized controlled trial. Circulation: Cardiovascular Interventions 2021;14(6):e010280
Zeller [16]	Orbital atherectomy prior to drug-coated balloon angioplasty in calcified infrapopliteal lesions: rationale and design of a randomized, multicenter pilot study (OPTIMIZE BTK). Journal of Endovascular Therapy 2018;25(Suppl 1):Abstract
	Orbital atherectomy prior to drug-coated balloon angioplasty in calcified infrapopliteal lesions: a randomized, multicenter pilot study (OPTIMIZE BTK). Journal of Endovascular Therapy 2022;29(6):874–884
Shammas [13]	Jetstream atherectomy followed by paclitaxel-coated balloons versus balloon angioplasty followed by paclitaxel-coated balloons for the treatment of femoropopliteal artery disease: rationale and design of the JET-RANGER study. Cardiovascular Revascularization Medicine 2020;21(12):1598–1602
	Jetstream atherectomy followed by paclitaxel-coated balloons versus balloon angioplasty followed by paclitaxel-coated balloons: twelve-month exploratory results of the prospective randomized JET-RANGER study. Vascular Health and Risk Management 2022;18:603–615

Studies are listed in chronological order by year of publication

### Patient Demographics and Lesion Characteristics

Patient demographics, clinical presentation, and lesion characteristics are summarized in Tables 2 and 3. FP trials predominantly enrolled claudicants (Rutherford 2–3) with shorter lesions, whereas IFP trials enrolled patients with chronic limb-threatening ischaemia (Rutherford 4–6) and longer disease.

**Table 2:** Baseline study characteristics

Study ID	Year	Country	Funding source	Follow-up duration (months)	Males (%)	Males SD
Shammas [14] (CALCIUM 360)	2012	USA	Cardiovascular Systems Inc	12	BA: 60	0.77
					ATH + BA: 68	
Dattilo [10] (COMPLIANCE 360°)	2014	USA	Cardiovascular Systems Inc	12	BA: 36	NA
					ATH + BA: 28	
Zeller [11] (DEFINITIVE AR)	2017	Europe (Belgium, Germany, Poland, Switzerland)	Medtronic	12	BA: 69	0.68
					ATH + BA: 65	
Zeller [16] (OPTIMIZE BTK)	2022	Europe (Austria, Germany)	Cardiovascular Systems Inc	12	BA: 74	0.561
					ATH + BA: 81	
Cai [12]	2020	China	Beijing Municipal Administration of Hospitals	24	BA: 71	0.217
					ATH + BA: 82	
Rastan [15]	2021	Europe (Germany, Austria)	Medtronic	12	BA: 83	NA
					ATH + BA: 70	
Shammas [13] (JET-RANGER)	2022	USA	Boston Scientific	12	BA: 69	0.246
					ATH + BA: 65

**Table 3:** Lesion characteristics

		Rutherford						
Study	Arm	2	3	4	5	Lesion length (mm)	Lesion diameter (mm)	Calcification presence
Shammas [14]	OA + BA	0	0	12/25 (48%)	44% (11/25)	91	NA	NA
	BA	0	0	12/25 (48%)	44% (11/25)	99	NA	NA
Dattilo [10]	OA + BA	NA	NA	NA	NA	55.9	5.3 ± 0.9	NA
	BA	NA	NA	NA	NA	87.3	5.5 ± 0.8	NA
Zeller [11]	DA + DCB	27.1% (13/48)	70.8% (34/48)	8.32% (1/48)	0	112.3 ± 40.3 (48)	4.9 ± 1.1 (48)	34/48
	DCB	24.1% (13/54)	74.1% (40/54)	1.9% (1/54)	0	96.6 ± 40.9 (54)	4.9 ± 0.9 (54)	40/54
Cai [12]	DA + DCB	NA	34/45 (75.6%)	7/45 (15.6%)	4/45 (8.9%)	113 ± 66	5.1 ± 0.5	NA
	DCB	NA	32/49 (65.3%)	9/49 (18.4%)	8/49 (16.3%)	111 ± 63	4.9 ± 0.6	NA
Rastan [15]	DA + DCB	2 (5%)	12 (30%)	4 (10%)	22 (55%)	191.6 ± 101.6	2.6 ± 0.6	28/40
	DCB	1 (2.5%)	8 (20%)	9 (22.5%)	22 (55%)	160.8	2.4 ± 0.5	36/40
Zeller [16]	OA + DCB	0	10 (31.3%)	2 (6.3%)	20 (62.5%)	101.3 ± 72.8	2.3 ± 0.8 (N = 31)	29/29
	BA + DCB	0	8 (23.5%)	4 (11.8%)	22 (64.7%)	78.8 ± 61.0	2.4 ± 0.9 (N = 35)	32/32
Shammas [13]	JET + DCB	3/31 (6.5%)	25/31 (80.6%)	3/31 (9.7%)	1/31 (3.2%)	108.3 ± 42.6	5.1 ± 1.0	26/31
	PTA + DCB	0/16 (0%)	11/16 (68.8%)	5/16 (31.3%)	0/16 (0%)	112.1 ± 76.4	5.1 ± 1.0	11/16

### Risk of Bias Assessment

All seven trials were judged to be at high overall risk of bias (see Table 4), due to the inability to blind participants and operators. Five trials employed adequate randomization methods with prospective trial registration. The other trials had additional methodological limitations including unclear randomization methods and limited reporting detail.

**Table 4:** The risk of bias judgements across all five domains for each included trial

Study	D1: Randomization	D2: Deviations	D3: Missing Data	D4: Measurement	D5: Selection	Overall
Shammas [14]	*Some*	High	*Some*	**Low**	*Some*	High
Dattilo [10]	**Low**	High	**Low**	**Low**	**Low**	High
Zeller [11]	**Low**	High	**Low**	**Low**	**Low**	High
Zeller [16]	**Low**	High	*Some*	**Low**	**Low**	High
Cai [12]	*Some*	High	*Some*	*Some*	*Some*	High
Rastan [15]	**Low**	High	*Some*	**Low**	**Low**	High
Shammas [13]	**Low**	High	*Some*	**Low**	**Low**	High

Cochrane conventions: Bold indicates low risk, Italic indicates some concerns, and Underline indicates high risk of bias

*D1* Randomization process; *D2* Deviations from intended interventions; *D3* Missing outcome data; *D4* Measurement of the outcome; *D5* Selection of the reported result. Some Some concerns

### Meta-Analysis Results

#### Procedural Outcomes

Procedural outcomes were reported across the included trials with varying completeness. Technical success was reported in six trials (441 patients) and showed a non-significant trend towards higher technical success rates with ATH + BA versus BA alone (OR 3.24, 95% CI 0.98–10.72, *p* = 0.05), with substantial study heterogeneity (*I*^2^ = 78%). The need for Bailout Stenting (Fig. 4) was also reported in six trials (452 patients) where atherectomy was associated with reduced need for bailout stenting by 86% (OR 0.14, 95% CI 0.03–0.67, *p* = 0.01), although substantial heterogeneity was observed (*I*^2^ = 75%, *τ*^2^ = 2.62). Five of the six trials demonstrated effects favouring atherectomy with only Rastan [15] showing a neutral effect. Consistent with this finding, atherectomy also reduced the flow-limiting dissections (Fig. 5) by 71% (OR 0.29, 95% CI 0.10–0.84, *p* = 0.02); in the five trials (375 patients), it was reported in with low heterogeneity (*I*^2^ = 38%). In contrast, distal embolization (five trials) and arterial perforation (four trials) reported no significant difference between groups for embolization (OR 1.80, 95% CI 0.41–7.93, *p* = 0.44) or perforation (OR 1.77, 95% CI 0.34–9.36, *p* = 0.50).

#### Patency and Reintervention Outcomes

**Fig. 2:**
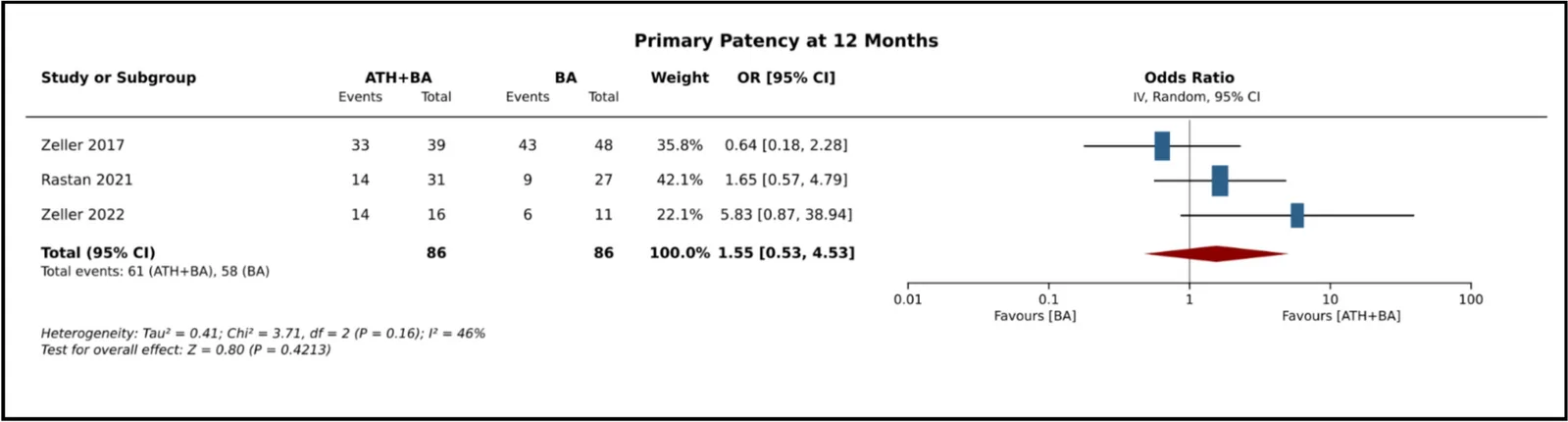
Forest plot of the combined results of primary patency at 12 months of relevant studies in ATH + BA group versus BA group alone

**Fig. 3:**
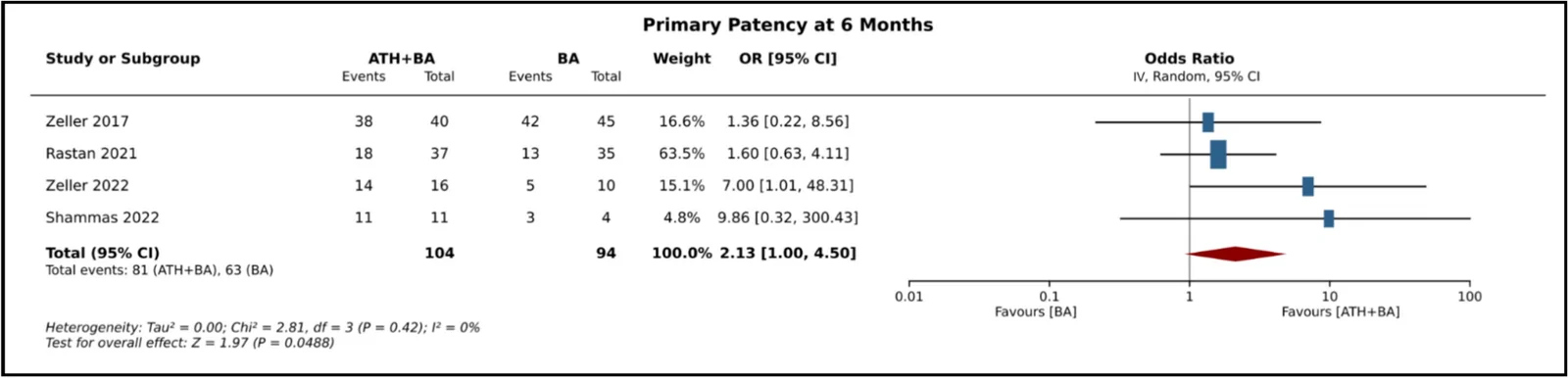
Forest plot of the combined results of primary patency at 6 months of relevant studies in ATH + BA group versus BA group alone

Across both patency and reintervention outcomes, statistically significant differences favouring atherectomy were not sustained at 12 months (Fig. 2) that was reported at 6 months (Fig. 3). Primary patency at 6 months was reported in four trials (198 patients) where the atherectomy showed an improvement in patency (OR 2.13, 95% CI 1.00–4.50, *p* = 0.049), with no observed heterogeneity (*I*^2^ = 0%).

By 12 months, three trials (172 patients) observed no significant differences in patency outcomes (OR 1.55, 95% CI 0.53–4.53, *p* = 0.42), with moderate heterogeneity (*I*^2^ = 46%). CD-TLR followed a similar trajectory at 6 and 12 months across all seven trials (474 patients). Atherectomy use was associated with a significant reduction in 6-month CD-TLR (OR 0.24, 95% CI 0.06–0.96, *p* = 0.04), with substantial heterogeneity (*I*^2^ = 64%). By 12 months, this statistically significant effect was no longer observed (OR 0.54, 95% CI 0.23–1.26, *p* = 0.16), with moderate heterogeneity (*I*^2^ = 44%).

#### Safety Outcomes

The two prespecified clinical safety endpoints were limited by low event rates across included trials. Major amputation at 12 months reported in five trials (348 patients) was reported only once in each arm, with no significant difference (OR 1.00, 95% CI 0.20–5.03, *p* = 1.00) and no heterogeneity (*I*^2^ = 0%). All-cause mortality at 12 months reported in six trials (407 patients) reported 12-month mortality with numerically more events in the BA group (17 events) compared with the ATH + BA group (7 events), but this difference did not reach statistical significance (OR 0.47, 95% CI 0.16–1.36, *p* = 0.16), with minimal heterogeneity (*I*^2^ = 14%). Both of these outcomes produced wide confidence intervals that preclude reliable inferences about between-group differences.

### Subgroup Analyses

Procedural advantages were greater in FP than IFP studies with larger reductions in bailout stenting (FP: OR 0.05, 95% CI 0.01–0.21; IFP: OR 0.86, 95% CI 0.31–2.40), flow-limiting dissections (FP: OR 0.22, 95% CI 0.08–0.56; IFP: OR 3.29, 95% CI 0.13–83.64), and technical success (FP: OR 5.18, 95% CI 1.23–21.79; IFP: OR 1.16, 95% CI 0.23–5.93, *P* = 0.18).

**Fig. 4:**
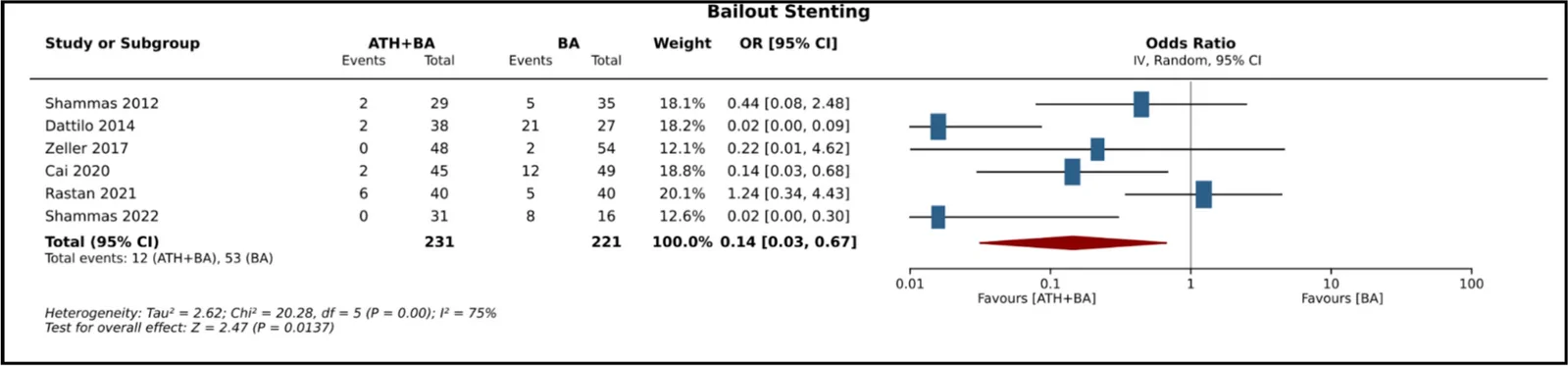
Forest plot of the combined results of bailout stenting of relevant studies in ATH + BA group versus BA group alone

Patency outcomes did not differ by anatomical location at 6 months; both subgroups had similar effect estimates (FP OR 2.12, 95% CI 0.42–10.78; IFP OR 2.61, 95% CI 0.67–10.13; interaction *P* = 0.85). At 12 months, the FP subgroup contained only a single study (Zeller [11]; OR 0.64, 95% CI 0.18–2.28), and the IFP subgroup included two studies (OR 2.41, 95% CI 0.77–7.49), precluding a formal interaction test. By anatomical location, no significant subgroup interactions were observed for CD-TLR at 6 months, CD-TLR at 12 months, all-cause mortality, or major amputation (Fig. 4).

**Fig. 5:**
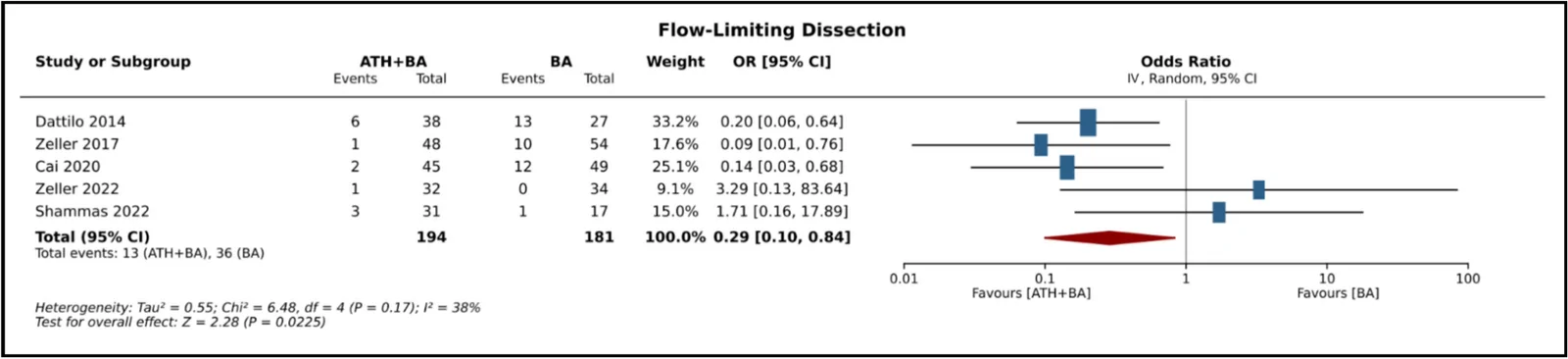
Forest plot of the combined results of flow-limiting dissection of relevant studies in ATH + BA group versus BA group alone

Subgroup analysis by adjunctive balloon type demonstrated a statistically significant interaction for 6-month CD-TLR (PBA: OR 0.05, 95% CI 0.01–0.17, 2 trials; DCB: OR 0.46, 95% CI 0.11–1.97; P-value = 0.021). For all other outcomes, no significant subgroup interaction by balloon type was identified. Subgroup estimates for all outcomes by lesion location and balloon type are presented in the supplementary material (Fig. 5).

## Discussion

This systematic review and meta-analysis demonstrates that atherectomy as vessel preparation prior to BA provides acute procedural benefits, including markedly reduced rate of bailout stenting by 86% and flow-limiting dissections by 71%, with improvements in 6-month primary patency. Avoiding provisional stenting is desirable, given that mechanical stresses in the FP segment predispose stents to fracture and subsequent in-stent restenosis [17], and plaque modification creates a more favourable substrate for subsequent balloon angioplasty by reducing vessel trauma. These procedural advantages appear to extend into improved 6-month primary patency and a significant reduction in CD-TLR at 6 months, though these benefits were not sustained at 12 months nor did they confer statistically significant effects for major amputation or mortality. It is worth noting that substantial between-study heterogeneity was observed for bailout stenting (*I*^2^ = 75%) and 6-month CD-TLR (*I*^2^ = 64%), reflecting variation in atherectomy device, lesion location, calcification severity, and adjunctive balloon strategy. A random-effects model was specified a priori to accommodate this. For bailout stenting, the direction of effect was consistent across trials, with five of six favouring atherectomy. For 6-month CD-TLR, the direction of effect varied between studies and a significant interaction by adjunctive balloon type was identified on subgroup analysis.

The divergence in outcomes between improved procedural and 12-month outcomes may be explained by the trials being predominantly pilots with small sample sizes, and the influence of atherosclerotic progression, neointimal hyperplasia, and vessel remodelling on long-term outcomes.

Pooled estimates for major amputation and all-cause mortality showed no between-group difference, but wide confidence intervals cannot exclude clinically meaningful effects in either direction. We retained these outcomes because they matter most to patients and clinicians, but the analyses are hypothesis generating only and require adequately powered trials with longer follow-up.

The prespecified subgroup analyses by lesion location indicate procedural advantages (reduced bailout stenting, flow-limiting dissections and increased technical success) favouring the FP group. This likely reflects two factors: bailout stenting is used selectively rather than routinely in IFP disease, where operators typically accept suboptimal angiographic results [18], and the FP trials enrolled more heavily calcified lesions [10, 12] that are well suited to debulking. The IFP subgroup contained only three trials, with single-study comparisons for flow-limiting dissection and 12-month patency, so these observations remain hypothesis-generating.

Two trials [10, 14] used plain balloon angioplasty, and the remaining five used drug-coated balloons, likely reflecting contemporary shifts in practice towards DCB. Subgroup analysis by balloon type was not feasible for 6- or 12-month patency, as no PBA trials reported patency outcomes. A statistically significant interaction was identified for 6-month CD-TLR, reflecting PBA trials counted bailout stenting within their composite reintervention endpoints, while the DCB trials reported clinically driven TLR separately. No significant interaction was identified for other outcomes.

Our findings are broadly consistent with previous systematic reviews examining atherectomy for PAD. The recent Cochrane review by Pherwani [19] concluded that evidence for atherectomy in PAD remains low regarding clinical effectiveness. The meta-analysis by Yiu et al. [20] also found no significant differences between atherectomy and other preparation methods.

Several limitations apply. First, the total sample of 489 patients limits power to detect modest effects, particularly for amputation and mortality. Second, prespecified subgroup analyses are constrained by small numbers of trials per subgroup and should be considered hypothesis-generating. Third, our PICO required matched balloon types between arms and therefore did not address ATH + BA versus primary stenting, an important contemporary comparison.

## Conclusion

Atherectomy as vessel preparation prior to balloon angioplasty significantly reduces bailout stenting and flow-limiting dissections compared with balloon angioplasty alone, with improved 6-month patency and reduced 6-month target lesion revascularization. However, these procedural benefits have not been shown to translate into significant improvements in 12-month clinical outcomes. The current evidence base is insufficient to support routine use of atherectomy for peripheral arterial disease. Larger, adequately powered trials with longer follow-up are needed to determine whether the acute procedural advantages of atherectomy confer durable clinical benefit for patients with FP and IFP atherosclerotic disease.

## Supplementary Information

Below is the link to the electronic supplementary material.

Supplementary file1 (DOCX 6708 KB)
