# Supplementary material for: Paving the Way: a Systematic Review and Meta-Analysis of Randomized Controlled Trials of Atherectomy as Vessel Preparation Prior to Balloon Angioplasty in Peripheral Arterial Disease
Source: Cardiovasc Intervent Radiol. 2026 Jul 13;49(9):1657–66. doi: 10.1007/s00270-026-04527-y (PMC13597600; doi:10.1007/s00270-026-04527-y)
Supplement: Supplementary file 1 [file 270_2026_4527_MOESM1_ESM.docx]

**Supplementary Table 1**

**CENTRAL (37)**

Last Saved:	20/09/2025 22:16:29

ID	Search

#1	MeSH descriptor: [Arteriosclerosis] this term only

#2	MeSH descriptor: [Arteriolosclerosis] this term only

#3	MeSH descriptor: [Arteriosclerosis Obliterans] this term only

#4	MeSH descriptor: [Atherosclerosis] this term only

#5	MeSH descriptor: [Arterial Occlusive Diseases] this term only

#6	MeSH descriptor: [Intermittent Claudication] this term only

#7	MeSH descriptor: [Ischemia] this term only

#8	MeSH descriptor: [Peripheral Vascular Diseases] explode all trees

#9	MeSH descriptor: [Vascular Diseases] this term only

#10	MeSH descriptor: [Leg] explode all trees

#11	MeSH descriptor: [Femoral Artery] explode all trees

#12	MeSH descriptor: [Popliteal Artery] explode all trees

#13	MeSH descriptor: [Iliac Artery] explode all trees

#14	MeSH descriptor: [Tibial Arteries] explode all trees

#15	((atherosclero* OR arteriosclero* OR PVD OR PAOD OR PAD)):ti,ab,kw

#16	(((arter*) near (*occlus* or steno* or obstuct* or lesio* or block* or obliter*) )):ti,ab,kw

#17	((vascular) near (*occlus* or steno* or obstuct* or lesio* or block* or obliter*) ):ti,ab,kw

#18	((veno*) near (*occlus* or steno* or obstuct* or lesio* or block* or obliter*) ):ti,ab,kw

#19	((vein*) near (*occlus* or steno* or obstuct* or lesio* or block* or obliter*) ):ti,ab,kw

#20	((peripher*) near (*occlus* or steno* or obstuct* or lesio* or block* or obliter*) ):ti,ab,kw

#21	(peripheral near/3 dis* ):ti,ab,kw

#22	arteriopathic:ti,ab,kw

#23	(claudic* or hinken*):ti,ab,kw

#24	(isch* or CLI):ti,ab,kw

#25	dysvascular*:ti,ab,kw

#26	(leg near4 (obstruct* or occlus* or steno* or block* or obliter*) ):ti,ab,kw

#27	(limb near4 (obstruct* or occlus* or steno* or block* or obliter*) ):ti,ab,kw

#28	((lower near3 extrem*) near4 (obstruct* or occlus* or steno* or block* or obliter*) ):ti,ab,kw

#29	((aort* or iliac or femoral or popliteal or femoro* or fempop* or crural) near3 (obstruct* or occlus*) ):ti,ab,kw

#30	0 1 2 3 4 5 6 7 8 9 0 1 2 3 4 5 6 7 8 9 0

#31	MeSH descriptor: [Atherectomy] this term only

#32	atherect*:ti,ab,kw

#33	(SilverHawk or "Silver Hawk" ):ti,ab,kw

#34	Jetstream:ti,ab,kw

#35	(atheroablation or rotational or orbital):ti,ab,kw

#36	EV3:ti,ab,kw

#37	#32 or #33 or #34 #35 #36

#38	#30 AND #37 with Cochrane Library publication date Between Jan 2012 and Sep 2025

#39	#38

**Medline (875)**

Ovid MEDLINE(R) ALL <1946 to September 22, 2025>

1	arteriosclerosis/	56881

2	arteriolosclerosis/	200

3	arteriosclerosis obliterans/	4069

4	atherosclerosis/	51376

5	arterial occlusive diseases/	28790

6	intermittent claudication/	8737

7	ischemia/	57540

8	exp peripheral vascular diseases/	68207

9	vascular diseases/	40405

10	exp LEG/bs [blood supply]	25679

11	exp Femoral Artery/	31927

12	exp Popliteal Artery/	10940

13	exp iliac artery/	15225

14	exp tibial arteries/	1916

15	(atherosclero* or arteriosclero* or PVD or PAOD or PAD).ti,ab.	245114

16	(arter* adj (occlus* or steno* or obstuct* or lesio* or block* or obliter*)).ti,ab.	87739

17	(vascular adj (occlus* or steno* or obstuct* or lesio* or block* or obliter*)).ti,ab.	21077

18	(vein* adj (occlus* or steno* or obstuct* or lesio* or block* or obliter*)).ti,ab.	11280

19	(veno* adj (occlus* or steno* or obstuct* or lesio* or block* or obliter*)).ti,ab.	9781

20	(peripher* adj (occlus* or steno* or obstuct* or lesio* or block* or obliter*)).ti,ab.	2422

21	(peripheral adj3 dis*).ti,ab.	56690

22	arteriopathic.ti,ab.	176

23	(claudic* or hinken*).ti,ab.	13134

24	(isch* or CLI).ti,ab.	505704

25	(leg adj4 (obstruct* or occlus* or steno* or block* or obliter*)).ti,ab.	747

26	(limb adj4 (obstruct* or occlus* or steno* or block* or obliter*)).ti,ab.	2759

27	(lower adj3 extrem* adj4 (obstruct* or occlus* or steno* or block* or obliter*)).ti,ab.	1953

28	((aort* or iliac or femoral or popliteal or femoro* or fempop* or crural) adj3 (obstruct* or occlus*)).ti,ab.	11900

29	dysvascular*.ti,ab.	349

30	or/1-29	1002345

31	ATHERECTOMY/	1041

32	atherect*.ti,ab.	4338

33	(SilverHawk or "Silver Hawk").ti,ab.	73

34	Jetstream.ti,ab.	77

35	(plaque adj3 excis*).ti,ab.	272

36	(atheroablation or rotational or orbital).ti,ab.	142253

37	(angle adj3 blade*).ti,ab.	231

38	(cut adj3 blade*).ti,ab.	127

39	(blade adj3 cathet*).ti,ab.	11

40	EV3.ti,ab.	281

41	or/31-40	145795

42	30 and 41	4689

43	randomized controlled trial.pt.	653402

44	controlled clinical trial.pt.	95754

45	randomized.ab.	728953

46	placebo.ab.	265979

47	drug therapy.fs.	2895289

48	randomly.ab.	478783

49	trial.ab.	797845

50	groups.ab.	2979384

51	or/43-50	6532015

52	exp animals/ not humans.sh.	5417661

53	51 not 52	5734248

54	42 and 53	885

55	limit 54 to yr="1860 - 2025"	875

**Embase (660)**

Embase <1974 to 2026 September 22>

1	exp atherectomy/	8861

2	(atherectom* or (rotational adj3 atherectom*) or TEC or Silverhawk or Turbohawk or Diamondback or "directional coronary atherectomy").ti,ab.	14860

3	1 or 2	17658

4	exp peripheral arterial disease/	83218

5	(peripheral adj3 (arter* or vascul*) adj3 disease).ti,ab.	57289

6	(PAD or PVD or "peripheral occlusive disease" or "lower limb ischaemia").ti,ab.	63578

7	4 or 5 or 6	152684

8	randomized controlled trial/	1138420

9	controlled clinical trial/	461304

10	random*.ti,ab.	2571863

11	placebo*.ti,ab.	476882

12	allocat*.ti,ab.	266695

13	trial*.ti,ab.	2458722

14	groups.ti,ab.	4309978

15	8 or 9 or 10 or 11 or 12 or 13 or 14	7542863

16	3 and 7 and 15	689

17	arteriosclerosis/	28972

18	arteriolosclerosis/	1299

19	peripheral occlusive artery disease/	59967

20	atherosclerosis/	196555

21	peripheral occlusive artery disease/	59967

22	intermittent claudication/	12086

23	exp femoral artery/	47114

24	exp popliteal artery/	15341

25	exp iliac artery/	88059

26	exp tibial artery/	5477

27	(atherosclero* or arteriosclero* or PVD or PAOD or PAD).ti,ab.	352196

28	(arter* adj3 (occlus* or steno* or obstuct* or lesio* or block* or obliter*)).ti,ab.	161675

29	(vascular adj3 (occlus* or steno* or obstuct* or lesio* or block* or obliter*)).ti,ab.	41129

30	(vein* adj3 (occlus* or steno* or obstuct* or lesio* or block* or obliter*)).ti,ab.	22320

31	(veno* adj3 (occlus* or steno* or obstruct* or lesio* or block* or obliter*)).ti,ab.	25933

32	(peripher* adj3 (occlus* or steno* or obstruct* or lesio* or block* or obliter*)).ti,ab.	30189

33	(peripheral adj3 dis*).ti,ab.	88751

34	arteriopathic.ti,ab.	219

35	(claudic* or hinken*).ti,ab.	19271

36	dysvascular*.ti,ab.	419

37	(leg adj4 (obstruct* or occlus* or steno* or block* or obliter*)).ti,ab.	1120

38	(limb adj4 (obstruct* or occlus* or steno* or block* or obliter*)).ti,ab.	4631

39	(lower adj3 extrem* adj4 (obstruct* or occlus* or steno* or block* or obliter*)).ti,ab.	2604

40	((aort* or iliac or femoral or popliteal or femoro* or fempop* or crural) adj3 (obstruct* or occlus*)).ti,ab.	16008

41	atherectomy/	5589

42	atherect*.ti,ab.	6961

43	(SilverHawk or "Silver Hawk").ti,ab.	147

44	Jetstream.ti,ab.	164

45	(plaque adj3 excis*).ti,ab.	580

46	(rotational adj3 atherectomy).ti,ab.	2682

47	(orbital adj3 atherectomy).ti,ab.	688

48	(angle adj3 blade*).ti,ab.	279

49	(cut adj3 blade*).ti,ab.	178

50	(blade adj3 cathet*).ti,ab.	16

51	EV3.ti,ab.	552

52	TurboHawk.ti,ab.	53

53	HawkOne.ti,ab.	19

54	Pantheris.ti,ab.	50

55	("Diamondback 360" or "Stealth 360").ti,ab.	60

56	(Phoenix adj3 atherectomy).ti,ab.	25

57	Rotarex.ti,ab.	170

58	Rotarex S.ti,ab.	22

59	Ocelot.ti,ab.	222

60	("Turbo-Elite" or "TurboBooster" or "Turbo-Tandem").ti,ab.	18

61	Clirpath.ti,ab.	6

62	("B-Laser" or Eximo).ti,ab.	113

63	("Pathway PV" or "Pathway Jetstream").ti,ab.	17

64	("Spectranetics" adj3 laser).ti,ab.	66

65	("CSI" adj3 orbital).ti,ab.	14

66	(Medtronic adj3 atherectomy).ti,ab.	8

67	(Avinger adj3 atherectomy).ti,ab.	5

68	(Philips adj3 atherectomy).ti,ab.	3

69	(AngioDynamics adj3 atherectomy).ti,ab.	5

70	41 or 42 or 43 or 44 or 45 or 46 or 48 or 49 or 50 or 51 or 52 or 53 or 54 or 55 or 56 or 57 or 58 or 59 or 60 or 61 or 62 or 63 or 64 or 65 or 66 or 67 or 68 or 69	11064

71	randomized controlled trial/	1138420

72	controlled clinical trial/	461304

73	random$.ti,ab.	2571863

74	randomization/	101823

75	placebo.ti,ab.	474417

76	(open adj label).ti,ab.	194448

77	((double or single or doubly or singly) adj (blind or blinded or blindly)).ti,ab.	375650

78	double blind procedure/	316937

79	parallel group$1.ti,ab.	54340

80	(crossover or cross over).ti,ab.	159674

81	((assign$ or match or matched or allocation) adj5 (alternate or group$1 or intervention$1 or patient$1 or subject$1 or participant$1)).ti,ab.	527922

82	(assigned or allocated).ti,ab.	627100

83	(controlled adj7 (study or design or trial)).ti,ab.	655540

84	trial.ti.	581514

85	(2019* or 2020* or 2021* or 2022* or 2023* or 2024* or 2025*).em.	45040437

86	17 or 18 or 19 or 20 or 21 or 22 or 23 or 24 or 25 or 26 or 27 or 28 or 29 or 30 or 31 or 32 or 33 or 34 or 35 or 36 or 37 or 38 or 39 or 40	827658

87	41 or 42 or 43 or 44 or 45 or 46 or 47 or 48 or 49 or 50 or 51 or 52 or 53 or 54 or 55 or 56 or 57 or 58 or 59 or 60 or 61 or 62 or 63 or 64 or 65 or 66 or 67 or 68 or 69	11064

88	71 or 72 or 73 or 74 or 75 or 76 or 77 or 78 or 79 or 80 or 81 or 82 or 83 or 84	3830342

89	86 and 87 and 88	660

**ClinicalTrials.Gov (264)**

peripheral arterial disease OR artery OR vascular OR Arteriosclerosis OR Is-

chemia | Atherectomy OR atheroablation OR rotational OR orbital OR EV3 |

| **Supplementary Table 1: Definitions of Primary and Secondary Outcomes** | |
| --- | --- |
| Outcomes | Definition |
| Technical Success | Successful treatment with a residual stenosis of less than 30% without bailout stenting. was defined as. was defined as. was defined as. was defined as. was defined as. was defined as. included |
| Primary patency | Freedom from restenosis greater than 50% (peak systolic velocity ratio ≥2.4 or ≥2.5 on duplex ultrasound) and freedom from target lesion revascularization |
| Bailout stenting | Unplanned stent placement for suboptimal angiographic result or flow-limiting dissection. Flow-limiting dissection was defined as grade C or higher dissection (as defined by NHLBI dissection classification) |
| Distal embolisation | Clinically significant embolisation to the distal vasculature requiring mechanical or pharmacological intervention |
| Arterial perforation | Vessel wall perforation |
| Clinically-driven target lesion revascularization (CD-TLR) | Any repeat endovascular intervention or surgical bypass of the target lesion prompted by clinical symptoms or hemodynamic deterioration (ankle-brachial index decrease ≥0.15 from post-procedure value) |
| Major amputation | Above-ankle amputation of the index limb |
| All-cause mortality | Death from any cause during the follow-up period. |

**Supplementary Figure 1: Forest plot of the combined results of Technical Success of relevant studies in ATH + BA group vs BA group alone**

**Supplementary Figure 2: Forest plot of distal embolisation rates in ATH + BA group vs BA group alone**

**Supplementary Figure 3: Forest plot of arterial perforation rates in ATH + BA group vs BA group alone**

**Supplementary Figure 4: Forest plot of the combined results of CD-TLR at 12 months of relevant studies in ATH + BA group vs BA group alone**

**Supplementary Figure 5: Forest plot of the combined results of Major Amputation at 12 months of relevant studies in ATH + BA group vs BA group alone**

**Supplementary Figure 6: Forest plot of the combined results of All-Cause Mortality at 12 months of relevant studies in ATH + BA group vs BA group alone**

**Femoropopliteal vs Infrapopliteal subgroup analyses**

**Supplementary Figure 7: Forest plot of primary patency at 12 months by lesion location in ATH + BA group vs BA group alone**

**Supplementary Figure 8: Forest plot of primary patency at 6 months by lesion location in ATH + BA group vs BA group alone**

**Supplementary Figure 9: Forest plot of Technical Success by lesion location in ATH + BA group vs BA group alone**

**Supplementary Figure 10: Forest plot of bailout stenting rates by lesion location (femoropopliteal vs infrapopliteal) in ATH + BA group vs BA group alone**

**Supplementary Figure 11: Forest plot of flow-limiting dissection rates by lesion location (femoropopliteal vs infrapopliteal) in ATH + BA group vs BA group alone**

**Supplementary Figure 12: Forest plot of distal embolisation rates by lesion location in ATH + BA group vs BA group alone**

**Supplementary Figure 13: Forest plot of arterial perforation rates lesion by location in ATH + BA group vs BA group alone**

**Supplementary Figure 14: Forest plot of the combined results of CD-TLR at 6 months by lesion location in ATH + BA group vs BA group alone**

**Supplementary Figure 15: Forest plot of the combined results of CD-TLR at 12 months by lesion location in ATH + BA group vs BA group alone**

**Supplementary Figure 16: Forest plot of major amputation at 12 months by lesion location in ATH + BA group vs BA group alone**

**Supplementary Figure 17: Forest plot of all-cause mortality at 12 months by lesion location in ATH + BA group vs BA group alone**

**Plain Balloon Angioplasty (PBA) vs Drug Coated Balloon (DCB) subgroup analyses**

**Supplementary Figure 18: Forest plot of Technical Success by adjunctive balloon type in ATH + BA group vs BA group alone**

**Supplementary Figure 19: Forest plot of bailout stenting rates by adjunctive balloon type in ATH + BA group vs BA group alone**

**Supplementary Figure 19: Forest plot of flow-limiting dissection rates by adjunctive balloon type in ATH + BA group vs BA group alone**

**Supplementary Figure 20: Forest plot of distal embolisation rates by adjunctive balloon type in ATH + BA group vs BA group alone**

**Supplementary Figure 21: Forest plot of arterial perforation rates by adjunctive balloon type in ATH + BA group vs BA group alone**

**Supplementary Figure 22: Forest plot of the combined results of CD-TLR at 6 months by adjunctive balloon type in ATH + BA group vs BA group alone**

**Supplementary Figure 23: Forest plot of the combined results of CD-TLR at 12 months by adjunctive balloon type in ATH + BA group vs BA group alone**

**Supplementary Figure 24: Forest plot of major amputation at 12 months by adjunctive balloon type in ATH + BA group vs BA group alone**

**Supplementary Figure 25: Forest plot of all-cause mortality at 12 months by adjunctive balloon type in ATH + BA group vs BA group alone**
